# Towards Novel Gene and Cell Therapy Approaches for Cervical Cancer

**DOI:** 10.3390/cancers15010263

**Published:** 2022-12-30

**Authors:** Robert Polten, Ivana Kutle, Jens Hachenberg, Rüdiger Klapdor, Michael Morgan, Axel Schambach

**Affiliations:** 1 Institute of Experimental Hematology, Hannover Medical School, 30625 Hannover, Germany; 2 Department of Obstetrics and Gynecology, Hannover Medical School, 30625 Hannover, Germany; 3 Division of Hematology/Oncology, Boston Children’s Hospital, Harvard Medical School, Boston, MA 02115, USA

**Keywords:** cervical cancer, immunotherapy, chimeric antigen receptor (CAR), T cell receptor (TCR), human papillomavirus (HPV), immune checkpoint inhibitors

## Abstract

**Simple Summary:**

Current treatments for cervical cancer patients include surgery, radiation and chemotherapy, which may be combined. Unfortunately, it is not always possible to remove all of the cancer and some cancer cells may be resistant to radio-/chemotherapy. This can result in lack of disease control or cancer relapse after initial response to therapy. Furthermore, metastasis of the tumor cells presents another difficult therapeutic challenge. The advent of novel immunotherapies, including monoclonal antibodies and genetically modified immune cells, that augment anti-cancer activity of immune cells holds great promise to improve cervical cancer patient survival.

**Abstract:**

Cervical cancer is one of the most common malignancies in women, and the majority of cases are caused by infection with high-risk human papilloma virus (HPV) subtypes. Despite effective preventative measures, such as vaccinations against HPV, over 300,000 women die world-wide from cervical cancer each year. Once cervical cancer is diagnosed, treatment may consist of radial hysterectomy, or chemotherapy and radiotherapy, or a combination of therapies dependent upon the disease stage. Unfortunately, overall prognosis for patients with metastatic or recurrent disease remains poor. In these cases, immunotherapies may be useful based on promising preclinical work, some of which has been successfully translated to the clinic. For example, approaches using monoclonal antibodies directed against surface proteins important for control of immune checkpoints (i.e., immune checkpoint inhibitors) were shown to improve outcome in many cancer settings, including cervical cancer. Additionally, initial clinical studies showed that application of cytotoxic immune cells modified to express chimeric antigen receptors (CAR) or T cell receptors (TCR) for better recognition and elimination of tumor cells may be useful to control cervical cancer. This review explores these important topics, including strengths and limitations of standard and developing approaches, and how some novel treatment strategies may be optimally used to offer the best possible treatment for cervical cancer patients.

## 1. Introduction

Cervical cancer is the fourth most common cancer in women, accounting for a yearly global incidence of approximately 600,000 cases. To date, most cervical cancer cases are associated with a lethal outcome, leading to around 340,000 deaths annually world-wide [1]. Cervical cancer initiates in cells that line the cervix, for example the glandular cells that cover the endocervix or the squamous cells that line the ectocervix. Approximately 75–90% of cervical cancers are squamous cell carcinomas, about 10–20% are adenocarcinomas that develop from glandular cells and there are also cases of adenosquamous cell carcinomas that are mixed carcinomas with features of both [2]. 

The description by Hippocrates in 400 BC of a disease that appeared to exclusively affect women may be the first written account of cervical cancer [3,4]. In the 1800s, the connection between sexual activity and cervical cancer became apparent, with cervical cancer even being considered a sexually transmitted disease. Early in the 1900′s, Wertheim described a radical hysterectomy approach that led to 5-year cancer-free status in more than 30% of cervical cancer patients [5]. Prevention of cervical cancer (e.g., by prophylactic vaccination) and detection of early pre-cancerous lesions are crucial factors to achieve favorable patient prognosis [6]. A milestone in the field of diagnostics was the development of a cytology smear test by Papanikolaou to analyze cells of the cervix with the objective to detect cancer cells [7]. This is now known as a Pap smear and was a precursor to the Pap test, which is now used as a screening test to detect early cervical cancer and its precursors.

**Figure 1:**
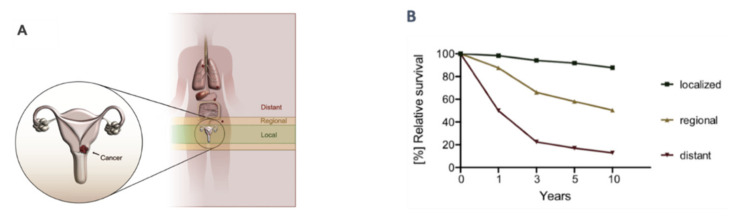
Overview of cervical cancer and relative survival rates in patients. (**A**) Cervical cancer can be broadly defined as local (confined to primary site), regional (spread to regional lymph nodes) or distant disease (distant metastasis). (**B**) The graph shows the relative 1-, 3-, 5- and 10-year survival of cervical cancer patients with respect to the tumor dissemination (data extracted from the SEER program (NIH)).

**Figure 2:**
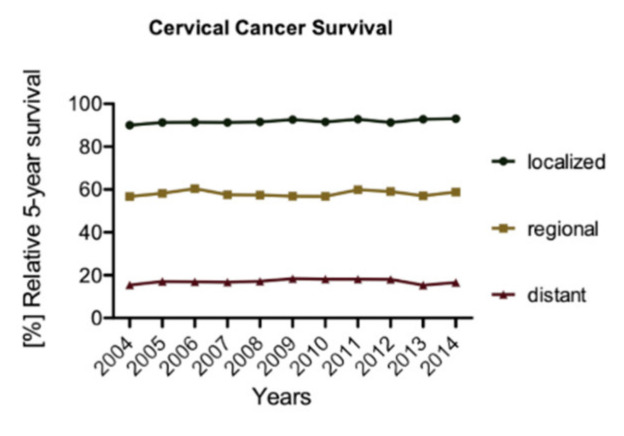
Summary of relative five-year survival from 2004 to 2014. Each data point corresponds to the percentage of patients alive five years after diagnosis (data extracted from the SEER program (NIH)).

As with many other diseases, the stage at which the patient presents can greatly influence choice of treatment, patient response and survival (Figure 1). Current treatment strategies for cervical cancer are based upon the type and stage of cancer, overall patient health, and, at least for early-stage disease, reproduction plans of the patient. Cervical pre-cancerous lesions can be treated with cryo or laser surgery to ablate the abnormal cells or conization to excise the affected tissue. Invasive cervical cancer can be removed by conization, simple or radical hysterectomy, trachelectomy or pelvic exenteration each with or without removal of nearby lymph nodes (e.g., sentinel lymph node dissection, or radial pelvic or paraaortic lymph node dissection). Standard of care for locally advanced cervical cancer is combined chemotherapy and radiotherapy (if available) or chemotherapy and hysterectomy in low-income areas [8]. Despite increased standard of care and improved surgical techniques, recurrence of cervical cancer remains a difficult therapeutic challenge and the 5-year survival rate of cervical cancer patients has not improved substantially over the last few decades (Figure 2).

Thus, there remains ample room to improve patient long-term survival and quality of life. In the following sections, we will discuss preventative measures such as vaccination to block human papillomavirus (HPV) dissemination, important pre-clinical and clinical advances with immune checkpoint inhibitors as immunotherapy approaches, adoptive cell therapies, including non-modified immune cells as well as chimeric antigen receptor (CAR)- or T cell receptor (TCR)-modified immune cells. Potential combinatorial treatment options will also be discussed and how these novel treatment modalities may contribute to development of new paradigms of cervical cancer patient care.

## 2. HPV Prevention Vaccine

As a most common sexually transmitted pathogen, HPV causes many conditions in men and women, including genital warts and even cancer [9]. HPV is a small, non-enveloped DNA virus that contains a circular, double-stranded viral genome and belongs to the Papillomaviridae family. The virus infects basal epithelial cells, primarily transmitted through contact to the skin or mucosa, for example by vaginal, anal and oral intercourse [10].

More than 400 distinct HPV types have been identified [11], of which 14 types are classified as oncogenic [12]. In the 1980′s, zur Hausen and Gissmann identified human papillomavirus strain 16 (HPV16) in pre-cancerous genital lesions and isolated HPV DNA from cancer cells [13]. These seminal discoveries led to a Nobel Prize in medicine for Harald zur Hausen in 2008 and directed the world-wide efforts to develop preventative vaccines, which have been instrumental in reducing cancer-causing infections as described below.

Cervical cancer accounts for almost 85% of all HPV-associated tumors [14], and HPV-16 and HPV-18 types are associated in 70% of all cases [15]. Besides cervical cancer, HPV infection is directly connected with developing anal, vulvar, vaginal, penis, and head and neck cancers [16]. Infections with HPV usually occur without clinical manifestations, the absence of local inflammations, and a weak humoral immune response. The development of progressive cancerous lesions from initial HPV infections is a long process divided into several phases: (i) infection of the cervical epithelium with high-risk human papillomavirus (hrHPV), (ii) non-clearance of HPV infection, (iii) uncontrolled proliferation of malignantly transformed infected cells, and (iv) invasion of malignant cells into the surrounding tissue [17,18]. 

In 1992, the discovery that the HPV capsid L1 protein assembles into non-infectious vector-like particles (VLPs) that completely reflect the structure of native HPV viral particles was a major breakthrough in the development of preventive HPV vaccines [19]. Further work on L1-based HPV vaccines resulted in the development of three worldwide available prophylactic vaccines that target hrHPV types, with limited cross-reactivity to closely related oncogenic HPV types and high efficacy, immunogenicity, and safety profiles [20,21,22]. The quadrivalent vaccine against HPV-6, HPV-11, HPV-16, and HPV-18 was authorized in 2006 (Gardasil^®^, Merck & Co., Rahway, NJ, USA); the bivalent vaccine against HPV-16 and HPV-18 in 2007 (Cervarix^®^, GlaxoSmithKline, Brentford. United Kingdom), and the nonavalent vaccine, against HPV-6, HPV-11, HPV-16, HPV-18, HPV-31, HPV-33, HPV-45, HPV-52, and HPV-58 in 2014 (Gardasil 9^®^, Merck & Co.) [23]. Generally, HPV preventive vaccines are applied in three doses in 6-month intervals to 9–14-year-old girls and boys. Over 200 million doses of prophylactic HPV vaccines have been administered worldwide since the implementation of HPV vaccination. Real-world data confirmed the effectiveness of vaccines in preventing HPV infection, with reduced prevalence of the two primary clinical outcomes: anogenital warts and high-grade cervical intraepithelial neoplasia (CIN) abnormalities in young women [24,25,26].

While vaccination before the age of 17 has shown decreased incidences of cervical cancer [27], no clear evidence exists for decreased cervical cancer incidence and mortality worldwide. One of the main limitations of HPV preventive vaccination is inadequate population coverage and global vaccine administration. Based on the latest reports, only 8% of low-to-middle-income countries had introduced the HPV vaccine, compared with 71% in high-income countries [28]. Due to the high vaccine costs and lack of resources to implement efficient education, vaccination, and screening programs, the burden of HPV infections and associated malignancies in low-income countries is exceptionally high, with cervical cancer as the second most common cancer among women [1,29]. While vaccination can be a successful preventative measure if adequate coverage is achieved throughout the population, it needs to be administered before the onset of cervical cancer to be effective. However, several ongoing clinical studies investigate the therapeutic potential of HPV vaccination for patients identified with HPV or HPV-associated cancers [30]. The anti-cancer mechanism of the therapeutic HPV vaccination approach is to promote generation of anti-HPV-specific immune responses, such as induction of cytotoxic T cells that target HPV-positive tumor cells. Alternatively, other targeted treatment strategies that can be applied to cervical cancer patients are described below. 

## 3. Immune Checkpoint Inhibitors in the Treatment of Cervical Cancer

Precise or personalized medicine approaches that focus on immunotherapies for cancer treatments have expanded drastically in the last 10 years. Primary immunotherapy principles are based on understanding how tumors manipulate and evade immunological responses. Lately, multiple compartments of the immune response are actively investigated to stimulate the immune system against tumors by administration of cancer vaccines, oncolytic viruses, immune checkpoint inhibitors (ICIs) or adoptive cell therapy (ACT) [31]. One of the greatest achievements in cancer treatment in the last decade has undoubtedly been the introduction of immunomodulators designed to block the immune checkpoints. The discovery of immune checkpoint inhibitors by James P. Allison and Tasuku Honjo was awarded with the Nobel Prize in Physiology or Medicine in 2018. Immune checkpoint inhibitors (ICIs) are usually monoclonal antibodies that induce the release of immune-suppressing brakes by targeting cytotoxic T-lymphocyte-associated protein 4 (CTLA-4), the programmed death 1 (PD-1) receptor or PD-1 ligands programmed death ligand 1 (PD-L1) and programmed death ligand 2 (PD-L2) [32]. Use of immune checkpoint inhibitors in anticancer therapies has rapidly expanded since the first monoclonal antibody against CTLA-4, ipilimumab, was authorized in 2011. Currently, multiple monoclonal antibodies against PD-1, PD-L1, CTLA-4, and LAG-3 immune checkpoint molecules have been extensively tested in clinical trials with encouraging results [33]. Available antibodies targeting PD-1 (pembrolizumab and nivolumab) and PD-L1 (atezolizumab and durvalumab) have become some of the most widely prescribed anticancer therapies used as single agents or in combination with chemotherapies to treat about 50 different types of cancer [34]. 

Early-stage cervical cancers can be efficiently controlled with standard treatments, such as radical surgery and chemotherapy. However, patients with high-risk locally advanced or recurrent/metastatic disease have a poor prognosis after standard treatments. The elevated rates of PD-L1 expression in up to 80% of cervical cancers, and its rare detection in normal cervical tissue, even when adjacent to CIN or cancer cells, support a strong rationale for the use of ICIs to restore the immune response against cervical cancer [35,36,37]. In the last few years, multiple clinical trials were designed to study the therapeutic effect of ICIs in cervical cancer in all clinical settings of localized, advanced and recurrent diseases. In 2018, pembrolizumab was the first immunotherapy approved by the US Food and Drug Administration (FDA) as a second-line treatment for patients with PD-L1-positive persistent or recurrent/metastatic cervical cancer. In 2021, the FDA approved two additional drugs: the antibody–drug conjugate tisotumab vedotin for second-line recurrent/metastatic disease [38] and the antibody pembrolizumab in combination with chemotherapy in the presence or absence of bevacizumab for first-line treatment of PD-L1-positive persistent or recurrent/metastatic disease [39,40,41]. In a clinical study (NCT03257267) of 608 advanced cervical cancer patients that compared PD-1 inhibitor cemiplimab monotherapy to chemotherapy, improved overall response (16.4% vs. 6.3%) and median overall survival (12 vs. 8.5 months) were observed for the cemiplimab group [42]. Currently, there are more than 20 additional immunotherapies in clinical trials with the potential for regulatory consideration, suggesting that ICI-based treatment strategies might represent a new standard of care for cervical cancer [43,44]. However, discovery of new biomarkers to identify the patients most likely to have significant benefits from these drugs will be an important next step to improve cancer patient outcomes with ICI therapy. Interestingly, the gut microbiome might be an important factor that influences therapy outcomes of patients treated with ICIs. The composition and relative abundance of certain bacterial strains within the microbiome was reported to be linked with different ICI treatment outcomes [45,46]. In a mouse model, presence of intestinal *Bifidobacterium* was associated with improved anti-tumor effects of ICIs, due to enhanced activation of dendritic and cytotoxic CD8^+^ T cells [47]. One approach is to utilize these findings in fecal microbiota transplantation (FMT), in which the microbiome of healthy or ICI-responding patients is transplanted to modify the microbiome of cancer patients [48]. Moreover, treatment of cervical cancer patients with *Lactobacillus acidophilus* and *Bifidobacterium* reduced the incidence of radiation-induced diarrhea [49], with FMT being evaluated as potential treatment for ICI-induced diarrhea and a current phase I clinical trial (NCT04038619). Another important consideration is how to increase access and affordability of ICI therapies so that patients in developing countries can also benefit from these advancements in cancer therapy.

## 4. Cell-Based Therapies

The promising results of immune checkpoint inhibitor therapy are offset by the occurrence of side effects, which may be severe, caused by the non-specific activation of the immune system [50]. Furthermore, only a subset of cancers responds and ICI treatment does not allow for specific elimination of tumor cells not previously recognized by the immune system. Similarly, immune therapy with cytokines only leads to an unspecific stimulation of the immune system and can be associated with severe side effects. In contrast, the cellular immune system eliminates tumor cells via specific and non-specific mechanisms and represents one of the most important components of effective anti-tumor therapy [51]. As compared to antibody therapy, cytotoxic immune cells, such as T or natural killer (NK) cells, can not only specifically eliminate tumor cells, but also modulate the local immune system and contribute to persistent tumor eradication [52]. The large number of different immune cells, such as dendritic cells, various subsets of T cells, NK cells or macrophages, gives rise to a wide variety of options for cancer therapy [53,54]. A few therapeutic strategies have already shown their benefit in pre-clinical experiments and have been translated into the clinical routine for different tumor entities [55,56,57]. There are currently no clinically approved cell therapy approaches to treat cervical carcinoma. Yet, cellular immunotherapy may have numerous advantages over non-specific chemotherapy, radiotherapy, ICI, or antibody-based immunotherapy. For example, cytotoxic immune cells, such as T or NK cells, can be engineered to recognize target antigens preferentially expressed on tumor cells and, thus, allow specific elimination of cancer cells that express the antigen(s). The highly specific re-targeting of immune cells to kill tumor cells can reduce possible side effects by the rather unspecific killing or immune cell activation that can occur with standard chemoradiotherapy or use of ICI. Additionally, immune cells can achieve long-term persistence, which may allow long-lasting tumor surveillance and control. However, some so-called “cold” tumors are surrounded by a tumor microenvironment (TME) that inhibits immune cell infiltration and are therefore less likely to respond to immunotherapy. Here, prediction tools can be used to classify cervical cancer based on their immune infiltration profiles to guide clinical treatment decisions [58]. Novel strategies in development show that immune cells can be genetically modified to overcome tumor resistance mechanisms and modulate the tumor microenvironment (TME) to achieve more effective, long-term antitumor responses [52].

Adoptive cell therapy (ACT) is one of the most promising approaches in cellular immunotherapy, in which autologous or allogeneic immune cells are collected, expanded, genetically modified if necessary, and re-administered to the patient. The following approaches are mainly used: (i) collection, expansion and, if necessary, modification of antigen-presenting cells (APC) to present tumor-specific antigens and stimulate the immune system. (ii) Isolation and expansion of endogenous tumor-specific T cells, including tumor-infiltrating lymphocytes (TILs). These can be isolated from different sources, such as the peripheral blood of patients or directly from the tumors. (iii) Modification of T and other immune cells using chimeric antigen receptors (CAR). CARs can be directed against tumor-associated antigens and lead to an activation of the respective immune cells upon binding to the targeted antigen in a major histocompatibility complex (MHC)-independent manner. (iv) Modification of T cells with tumor-specific TCR. This is intended to generate tumor-specific T cells, but engineered T cells can only be administered to human leukocyte antigen (HLA) compatible patients. These concepts will be explored more deeply in the following paragraphs.

### 4.1. Adoptive T Cell Therapy with Unmodified Cells

Despite modern chemotherapy, advanced and metastatic cancers represent a major challenge in oncology. Increased development of individualized, patient-specific therapies is expected to result in better efficacy and reduced unwanted side effects such as toxicity to healthy tissue. Already in 1900, Paul Ehrlich formulated the idea of the magic bullet, a therapy with 100% effect and no off-target activity [59]. The hypothesis that the immune system plays a decisive role in cancer development and therapy was formulated by Virchow in 1863, when he proposed that cancer is the product of unresolved inflammation [60]. Chronic inflammation has since been shown to play an important role in at least 25% of all oncological diseases [61,62,63].

However, delivery of immune cells as donor lymphocyte infusions (DLI) or complete replacement of the immune system have also been successfully used in treating cancer. In addition to established methods such as stem cell transplantation in treating various types of leukemia, squamous cell carcinoma of the bladder, for example, was successfully treated by vaccination with an attenuated *Mycobacterium bovis* strain and the resulting inflammation [64]. Allogeneic hematopoietic stem cell transplantation (HSCT) represents the earliest adoptive transfer of T cells immunologically directed against cancer cells. Here, the donor cells mediate a graft-versus-tumor effect directed against allogeneic antigens of the leukemia cells [65]. However, the undesirable side effect of the graft-versus-host disease (GVHD) remains a major problem. Consequences may include hepatosplenomegaly, atrophy of lymphoid organs, diarrhea, skin changes, cachexia and even death of the patient. One strategy to reduce the risk of severe GVHD is the practice of T cell depletion. However, the rate of disease recurrence also increased, demonstrating the importance of an immune response for successful antitumor therapy [66].

### 4.2. Natural Tumor-Infiltrating Lymphocytes

Tumor-infiltrating lymphocytes (TILs) have been described for many different cancers, and high levels of TILs are associated with favorable prognostic outcome. This was previously shown for melanoma, breast cancer, lung cancer, colorectal cancer, kidney cancer, prostate cancer and gastric cancer [67,68,69,70,71,72,73,74,75]. TILs are typically organized as clusters and critical factors that can influence their anti-cancer functionality are localization and differentiation. T cells are divided into CD4^+^ and CD8^+^ depending on their T cell receptor subunit. Here, the αβ-TCR complex is distinguished from the γδ-TCR subunit. While the αβ-subunit is important for recognizing surface peptides together with the MHC class I (CD8^+^ T cells) or class II (CD4^+^ T cells), the γδ-subunit functions independently of MHC [76]. In patients with early-stage cervical carcinoma, the absence of lymphogenic metastasis was associated with increased CD8^+^/CD4^+^ ratios and CD8^+^/regulatory T cells. In these small cohorts, 5-year survival was shown to be significantly different at 92% vs. 73%, with higher numbers of TILs associated with better prognosis [77,78]. However, this beneficial effect is not limited to early cervical carcinoma. TILs were also shown to have a beneficial effect in advanced cervical carcinoma as distinct infiltration of CD3^+^, CD4^+^, CD8^+^, CD206^+^ and FOXP3^+^ TILs were significantly associated with progression-free and overall survival in cervical cancer [79]. Additionally, it was shown that irradiation of colon tumor cells led to an increased expression of MHC class I, which induced enhanced CD8^+^ T cell recognition of tumor cells and reduced immune evasion [80]. 

### 4.3. Alternative Cell Sources for Immune Therapy

APC are a group of immune cells that process antigens (e.g., tumor-derived antigens) and present their peptide fragments on the cell surface via MHC molecules. Dendritic cells (DC) are a type of APC that can, upon encounter with a target antigen, migrate to lymph nodes and present antigens to other immune cells. Importantly, dendritic cells are capable of cross-presentation, a process that allows the presentation of exogenous tumor antigens and MHC class I molecules, thus allowing them to prime CD8^+^ T cells. Besides their function as APCs, DCs also contribute to the immune response by releasing chemokines, such as CXCL10, thereby further enhancing the anti-tumor effects of immune cells [81,82]. Their central role in the immune system and their ability to alter the TME make DCs an interesting cell type for immunotherapy [83]. DCs were successfully used as a cell-based vaccine, where DC precursors are extracted from the patient and equipped with a distinct antigen that will be presented by the DC after reinfusion into the patient. An autologous DC therapy against prostate cancer was the first FDA-approved cellular therapy [50]. Due to the common association of cervical cancer to HPV infections, this cancer entity might be of particular interest for the development of cancer vaccines and are currently evaluated in clinical trials [84]. A dendritic cell vaccination strategy using HPV16 E7 peptides has shown promising results for targeting of cervical cancer cells in an in vivo murine model [85].

Like dendritic cells, macrophages are APCs that sense their environment and can engulf pathogens and apoptotic cells [86]. Classically, macrophages are broadly classified into proinflammatory M1- and anti-inflammatory M2-macrophages and can, based on their relative abundance, influence the TME. For example, a TME in which the predominant macrophage population consists of M2-polarized, immunosuppressive macrophages, also known as tumor-associated macrophages (TAM), is associated with a poor prognosis [87,88]. Moreover, TAMs are associated with angiogenesis and metastasis in multiple tumor entities, including cervical cancer [89,90]. Cervical cancer cells were reported to secrete PGE_2_ and IL-6, leading to decreased dendritic cell differentiation and a shift towards M2-polarized macrophages [91]. Therefore, strategies were developed to eliminate TAMs or re-polarize M2 into M1 macrophages [92]. Interestingly, irradiation of cervical cancer was described to be associated with a shift towards M1 macrophages, potentially mediated by extracellular vesicles [93]. In another approach to remodel the TME, macrophages were genetically engineered to secrete particular pro-inflammatory cytokines (e.g., IL-12), resulting in activated immune cell responses [94].

NK cells do not require exposure to antigens to mediate their anti-tumor properties [95] and target recognition is MHC-independent [96], an important characteristic for tumor diseases in which MHC loss leads to tumor progression and immune evasion [97,98]. That NK cells play such an important role in immune surveillance is further evidenced by the fact that lower NK cell activity was associated with increased cancer susceptibility and metastasis probability [99,100,101]. In addition to their cytotoxic function, NK cells also exhibit immunoregulatory functions by producing cytokines and chemokines that influence the function of B lymphocytes, T lymphocytes, dendritic cells, macrophages and neutrophils [102]. Functional MHC class I molecules on healthy cells bind inhibitory immunoglobulin-like killer cell receptors (KIR), which leads intracellular signals that reduce NK cell cytotoxic activity. Tumor cells that down-regulate MHC class I molecules to evade immune responses, e.g., by cytotoxic T cells, are thus recognized and eliminated by NK cells [103,104,105,106]. Additionally, NK cells can recognize antibody-coated cells through the FcγRIIIA (CD16) receptor and trigger antibody-dependent cellular cytotoxicity (ADCC) and cytokine production [107]. The cytotoxic effect of NK cells is triggered primarily via the release of cytolytic granules and cytotoxic cytokines [108]. For the antitumor efficacy of immune cells, the penetration into the tumor bed and the subsequent exposure of tumor cells to immune cells is a crucial step. This is particularly important in solid tumors where the TME is largely immunosuppressive. For example, in breast cancers, it was shown that increased release of the chemokine CX3CL1, which attracts NK cells, T lymphocytes, and dendritic cells, is associated with a better prognosis [109]. In addition, this also seems to influence the development of cancers. Thus, a substantial dysregulation of the CXCR3 and CXCR4 chemokine receptor/ligand axes was shown for multiple myeloma [110]. In 2017, a study reported high cytotoxic effects of umbilical cord blood-derived NK cells against cervical cancer and identified an enhanced cancer lysis compared to peripheral blood-derived NK cells [111,112].

### 4.4. Modification of Somatic Cells for Therapeutic Use

In cell therapy, genetic modification is a well-established approach to increase the endogenous anti-cancer activities of immune cells. Hereby, immune cells are isolated from patients (autologous cell therapy) or suitable donors (allogeneic cell therapy), genetically modified, expanded, and finally (re-)infused into the patient. With progress in biological engineering, many studies focused on developing gene modification and gene-delivery technologies. In this regard, retroviral vectors are commonly used for the long-term expression of gene cassettes, although retroviral vector-mediated gene transfer has an inherent risk of insertional mutagenesis [113]. To address such safety concerns, seminal advances have been made, including the use of self-inactivating (SIN) vectors and the switch from gammaretroviral to lentiviral vector systems, which preferentially insert into enhancer/promoter regions or actively transcribed genomic loci, respectively. Additionally, an alpharetroviral vector system that exhibits a relatively neutral integration pattern was developed [114,115,116]. Based on these technologies, multiple approaches have been conducted to redirect immune cells to target cancer cells, including cervical cancer cells. Here, targeting the viral-derived tumor-associated antigens might be an especially promising approach as most cervical cancer cells harbor HPV infections [117].

### 4.5. Chimeric Antigen Receptor-Based Cell Therapy

The above-mentioned genetic modification of immune cells is a promising approach to enhance endogenous anti-cancer effects by equipping immune cells with enhanced anti-tumor specificities, enabling the killing of cancer cells while leaving healthy tissue unharmed. Equipment of immune cells with CARs, first described in late 1987 by Kuwana et al. [118], enables the targeted recognition and binding of antigens expressed, for example, particularly by tumor cells. While this technology was originally developed in T cells, the approach has been extended to different immune cells, including NK cells and macrophages [119,120]. The structure of CARs consists of an extracellular antibody-derived single-chain variable fragment (scFv) linked through a transmembrane domain to an intracellular CD3ζ signaling domain. Activation of these components further activates downstream signaling pathways, resulting in cytotoxic effects against the target cell. Over two decades later, the core structure is still commonly retained, but has been expanded mainly by introduction of additional intracellular domains. Among these, coupling the CD3ζ signaling domain with either CD28 or 4-1BB co-stimulatory domains is the most commonly used design in market authorized CAR-T cell therapies. CD28 enhances cytotoxic activity, while 4-1BB is associated with an improved in vivo persistence of CAR-T cells [121]. Application of second-generation CAR-T cells was shown to be effective in clinical trials, especially against CD19-positive B cell lymphoma, frequently leading to complete tumor regression [122,123]. However, the clinical success of CAR-T cell therapy is mainly limited to hematological malignancies, and most CAR-T cells tested to date lack efficiency when employed as therapy against solid tumors. For successful transfer of this approach to treat solid cancers, especially the immunosuppressive TME and the exhaustion of CAR-T cells are major challenges that must be addressed by improved vector design [113,124].

As a result, the design of CAR vectors has been extended to include additional genetic information to deliver gene expression cassettes. These encode for cytokines that enhance the activity or persistence of CAR-T cells, or lead to the recruitment of additional immune cells. In 2017, Hu and colleagues generated CAR-T cells that were equipped with constitutive IL-18 expression, leading to enhanced CAR-T cell proliferation and anti-tumor activity in a murine xenograft model [125]. To restrict expression of cytokines to the local TME, inducible T cells redirected for universal cytokine killing (TRUCK) approaches were developed that allow expression of additional gene-cassettes exclusively upon binding of CAR-T cells to the target antigen, thereby limiting potential adverse effects of systemic delivery. Some studies used this approach to alter the immunosuppressive TME by the inducible expression of pro-inflammatory cytokines, including IL-2, IL-12, and IL-18, resulting in recruitment, stimulation and enhanced cytotoxic activity of surrounding immune cells [51,126,127]. Moreover, the inducible expression of IL-7 and IL-15 are currently being explored, which were reported to enhance the persistence of CAR-modified immune cells in vivo [128,129]. The choice of the inducible cytokine to be delivered to the TME is a critical parameter of such approaches, as different types of tumors may utilize distinct immune evasion strategies that need to be overcome. For example, evidence suggests that HPV may antagonize IL-18 function, which could result in inefficient immune mediated clearance of HPV-positive cervical cancer cells [130].

A multiple-component split CAR system was developed in which the CAR-T cell is selectively activated in the otherwise suppressive TME characteristic of the tumor entity [131]. Another strategy replaced the intracellular domain of PD-1 with a T cell co-stimulatory signal to switch the immunosuppressive signal into an activating signal [132]. To prevent the checkpoint-mediated inactivation, CAR-T cells have been modified to secrete scFvs that block the PD-1 receptor, hence preventing T cell exhaustion and prolonging in vivo persistence [133].

Heterogeneity of solid tumors is another major challenge to successful CAR therapy as this further complicates the selection of appropriate target antigens, as some antigens will be expressed only on subsets of cancer cells within the tumor. Thus, antigen-negative tumor cells may escape the CAR-T cells and lead to treatment failure (e.g., lack of response or relapse in case of initial response). Downregulation of the target antigen expression on cancer cells during the treatment is another critical point that can negatively impact the efficacy of CAR-T cell therapy. Therefore, the potential to react to changes in the protein expression profile of cancer cells is a crucial factor to consider for optimal therapy outcomes. One approach that maximizes the repertoire of target antigens is the concept of “universal” CARs. Here, the antigen-recognition domain of the CAR is separated from the signaling domain. The signaling domain is linked to an extracellular Avidin sequence, which is capable of binding to a Biotin molecule that is coupled to the respective scFv (antigen-recognition domain). This “lock-key” mechanism of universal CARs can theoretically be used to bind to an infinite number of antigens and may provide a solution to the tumor heterogeneity challenge [134,135].

It is not always possible to identify antigens that are exclusively expressed on cancer cells, with little to no expression on healthy cells. In the case that the targeted antigens are also widely expressed on healthy cells, collateral destruction of healthy tissues through the so-called “on-target off-tumor” effect may occur. Strategies to increase the selectivity of CAR therapy for tumor cells may help overcome these deleterious side-effects [124]. In this context, T cells simultaneously expressing two CARs with different antigen-specificities have been developed, which unfold their full cytotoxic activity solely in the presence of both tumor-associated antigens [136,137].

Currently, few clinical studies focus on CAR-based cell therapy against cervical cancer (Table 1). Two phase I clinical studies evaluate CAR-T cells redirected against CD22 (NCT04556669) or CD70 (NCT05518253), respectively. Another trial (NCT03356795) evaluates multiple targets for CAR-T therapy against cervical cancers, including GD2, PSMA, MUC1, and Mesothelin, but no results have been reported to date. Another study evaluated anti-Mesothelin-directed CAR-T cell therapy in a Phase I/II clinical trial (NCT01583686), but was terminated due to insufficient accrual. The clinical outcome of this study was less promising, as only one of the fifteen patients achieved a stable disease and no partial or complete responses were observed, but six serious adverse events were reported. 

### 4.6. TCR-Modified Cell Therapy 

A critical limitation of CAR therapy is the restriction to target antigens that are expressed on the surface of cancer cells. Therefore, intracellular proteins, which account for the largest fraction of cellular proteins, cannot be targeted by this approach. In contrast, TCRs can also bind to peptide fragments derived from intracellular proteins, but remain restricted to MHC-presented target structures. Like the CAR approach, TCRs have been genetically modified to bind tumor-associated peptide fragments that are presented on MHC complexes. These engineered TCRs structurally consist of α- and β-chains derived from T cell clones selected for their high target affinities. This engineered ligand binding site is linked to the endogenous TCR signaling components, including CD3δ, CD3ε, CD3γ, and CD3ζ domains [138,139]. Based on the higher number of subunits and co-receptors, TCR-engineered T cells were described to exhibit longer-lasting immune synapses with the target antigen compared to CAR-T cells [140]. Several modifications of the TCR complex were developed to utilize the advantages of the TCR signalling complex while gaining MHC independency. Among these are T cell receptor fusion constructs (TRuCs), T cell antigen couplers (TACs), and antibody-TCRs (AbTCRs), which are based on the fusion of antibody-derived recognition domains to the CD3 subunits of TCRs [141,142,143,144]. Moreover, immune mobilizing monoclonal T cell receptors against cancers (ImmTACs) were developed that combine soluble, affinity-enhanced TCRs (recognition domain) with a CD3-scFv effector domain [145]. Lastly, Walseng and colleagues combined the myriad target options of TCRs with the modularity of CARs by fusing a soluble TCR to the signalling domains of a CAR [146]. As over 90% of cervical cancers are associated with HPV infections [147], viral proteins are apparent targets for TCR-based therapies as they are absent in healthy cells. Especially HPV E6 and E7 oncoproteins are interesting target candidates as they show high expression levels in cervical cancer and exhibit high evolutional conservation [148,149]. In a preclinical study, engineered E7 TCR-T cells efficiently mediated the regression of HPV-positive tumors in mice [150]. Ongoing clinical studies evaluate the treatment of engineered TCR-T cells against the viral E6 (NCT03578406) and E7 (NCT02858310) oncoproteins (Table 2). Concerning the latter, an update was published last year, reporting that 6 of the 12 patients enrolled had a partial response and 4 patients had stable disease [151]. In another phase I/II clinical trial (NCT02280811) that focused on the evaluation of engineered E6 TCR-T cells, 2/12 patients had a partial response, and 4/12 patients had stable disease [152]. 

## 5. Combinatorial Therapies—Chemo-/Radiation Therapy

While genetic engineering of immune cells is already a promising approach to treat (cervical) cancer, a combination with other already clinically implemented strategies might lead to improved patient outcomes. A multitude of chemotherapeutics was shown to exert immunomodulatory effects and to sensitize tumor cells to immunotherapy, e.g., by increasing tumor infiltration or persistence of immune cells [153,154,155,156]. A combination of ErbB-targeting CAR-T cells and carboplatin treatment enhanced the therapeutic efficiency compared to monotherapy in an ovarian cancer xenograft mouse model [157]. Moreover, chemotherapy and radiotherapy were described to improve tumor antigen presentation, e.g., by enhanced MHC class I expression, in cervical cancer, breast cancer and multiple other tumor entities [156,158,159,160], which can be advantageous in combination with engineered TCR- or CAR-based therapy approaches. Cancer stem cells (CSCs) are often associated with improved chemoresistance [161,162], which might enhance tumor vulnerability to CSC-targeted therapy. Elimination of CSCs might be particularly important to overcome therapy resistance and tumor recurrence. It was recently shown that preconditioning ovarian cancer cells with cisplatin, followed by a CD133-targeted CAR therapy, led to efficient eradication of CSCs [163]. Another possibility is the combination of immune cell therapy with checkpoint inhibitors (e.g., Ipilimumab, Pembrolizumab) to decrease immune cell suppression. Several studies identified that chemotherapeutic treatments could increase PD-L1 expression on multiple tumor entities, including gynecologic malignancies [164,165]. A phase I clinical trial (NCT01711515) identified an increase in PD-1 expression in T cells following chemoradiation therapy in advanced cervical cancer [166]. A combination of chemoradiation with PD-1/PD-L1 inhibitors showed benefits in clinical trials for other tumor entities, including non-small-cell lung (KEYNOTE-189, NCT02578680), and is a promising approach for the treatment of cervical cancer. A phase II clinical trial (NCT02635360) currently examines the combinatorial treatment of chemoradiation and the PD-1 inhibitor pembrolizumab in cervical cancer patients. Subsequently, a combination of immune cell therapy with chemotherapy and checkpoint inhibition must be further evaluated.

## 6. Conclusions and Perspectives

**Figure 3:**
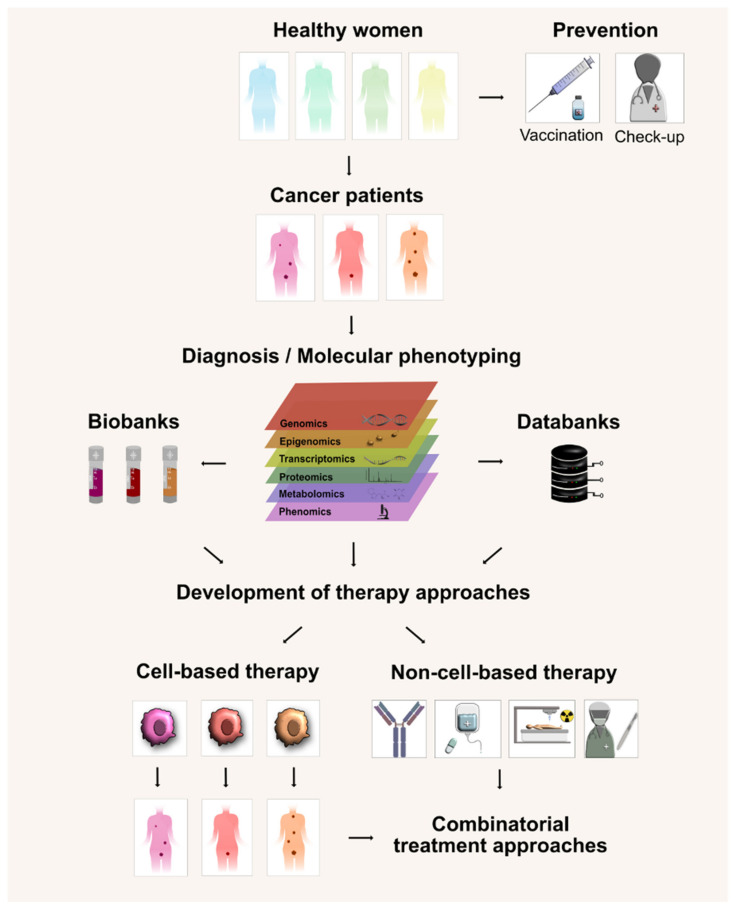
Perspectives of personalized anti-cervical cancer therapy. A high coverage of HPV vaccination and regular medical check-ups are required to prevent the development of cervical cancer. Upon diagnosis of cervical cancer, patient disease is categorized by staging systems and the molecular phenotypes are determined by multiple omics platforms. Tissue specimens/data are stored in biobanks/databanks and provide the basis to direct development of perspective therapy approaches. Based on the molecular profiling of the patient’s cells, personalized off-the-shelf cell-based therapy approaches (e.g., CAR- or TCR-modified immune cells) can be used either alone, or in combination with non-cell-based therapy approaches (e.g., antibodies/check-point inhibitors, chemo-radiation, surgery).

As discussed above, increased understanding of the disease biology and etiology has led to important preventative measures, such as vaccinations to help prevent cervical cancer due to HPV infection. Additionally, gynecological examinations to detect precancerous lesions or early disease stages offer women better health care with improved quality of life. Advances in “big data” fields are expected to further improve diagnostics by inclusion of molecular genotype and phenotype analyses that will help identify additional characteristics of cervical cancers. As part of The Cancer Genome Atlas (TCGA) project, a comprehensive study characterized cervical cancer on a genetic and molecular level and identified highly mutated genes (e.g., *ERBB3)* and pathways (e.g., PI3K/MAPK, TGFβ) as promising therapeutic targets [167]. Other studies uncovered major immunological differences between HPV-positive and HPV-negative cervical cancers, including differences in immune cell infiltration and tumor cell MHC-presentation [168,169]. This may also provide critical information necessary to choose best available patient treatment strategies as well as to direct development of new targeted therapy approaches (Figure 3).

There is accumulating evidence supporting the potential clinical usefulness of immunotherapeutic approaches including antibodies, CAR- and TCR-modified T and NK cells to treat solid tumors like cervical cancer [33,125,150,152]. Establishment of databanks with more completely characterized cervical cancer samples will help to further increase successful implementation of these treatment strategies. Beyond identification of novel tumor cell markers to target for cancer eradication, better markers for more precise and/or earlier diagnosis may be detected. In addition to protein markers, other biomolecules, including DNA and (mi)RNA (e.g., derived from liquid biopsies), are promising markers that can aid diagnosis, prognostics, and may be used in combination with novel prediction tools to guide therapeutic strategies [170,171]. Furthermore, such information could reveal inherent biological pathways that may be exploited to increase the rate of favorable patient outcome, for example, by decreased incidence of recurrent disease. The concept of the cancer stem cell is one hypothesis that has been raised to explain disease refractoriness or relapse [172]. Use of artificial intelligence to collate and examine multiple large data sets from cervical cancer tumors may reveal which cells within the tumor are cancer stem cells and whether or not a cancer stem cell hierarchy exists in cervical cancer. Here, it will be important to carefully characterize tumor cells at diagnosis and after treatment failure as well as the immune cells from patients whose tumors responded to treatment versus those whose cancer did not respond.

Elucidation of antigens more specifically expressed on cervical cancer (stem) cells will aid in development and application of precision immunotherapies, such as CAR-T/NK cells. Here, application of different immune cell combinations (e.g., T cells + NK cells + macrophages) to deliver an anti-cancer immunological armada may be more effective in generating the most potent anti-tumor activity. Perhaps combinatorial strategies such as initial tumor debulking with standard established methods (e.g., chemotherapy, radiology, surgery) and then application of CAR-modified cells when the tumor burden is low would lead to better disease control and elimination of micrometastases. Such approaches may help to alleviate recurrent disease and offer patients a longer disease-free remission.

Drawbacks of autologous cell therapies (unmodified or engineered cells) include that the process to generate them is patient- and disease-specific and may even exceed the time of the therapeutic window. Therefore, allogeneic approaches are increasingly gaining importance with the idea to use universally applicable therapeutic cells. Such cells can be provided from cell banks in a purified and tested form, which reduces some of the hurdles of the individual production necessary for autologous cells. Promising strategies use genetically modified stem cells from peripheral or cord blood, cell lines or iPSC (induced pluripotent stem cells). CRISPR-Cas9-mediated genome editing of iPSCs to disrupt the HLA locus resulted in generation of engraftable cell products with reduced immunogenicity [173,174]. Genome modification of T cells to knock-out the TCR is another common strategy implemented to generate off-the-shelf allogeneic CAR-T cell therapeutic products [175,176,177,178].

Another important aspect that should be considered is how to make promising immunotherapeutic approaches broadly available as costs may preclude use in lower income areas, where disease burden is often highest. Here, lower cost mobile/pre-fabricated production and distribution centers may be a feasible solution to consider, similar to the strategy followed by BioNTech to increase vaccine availability in Africa. While the prognosis for many cervical cancer patients is markedly better than the original description of cervical cancer as an untreatable disease by Hippocrates, ample opportunities remain to further improve outcome of these patients, especially for those with more advanced disease.

## Figures and Tables

**Table 1:** CAR-T cell-based clinical trials against cervical cancer.

Trial No. (Location)	Target	Condition	Phase	Patient No.	Start	Status	Results
NCT01583686 (United States, Maryland)	Mesothelin	Metastatic/unresectable Mesothelin-positive cancer	I/II	15	2012	Term. (2018)	CR: 0 PR: 0 SD: 1 PD: 14 SA: 6
NCT03356795 (China, Guangdong)	GD2, PSMA, MUC1, Mesothelin	Patients with stage III, IV or relapsed cervical cancer	I/II	20 (est.)	2017	Pending	
NCT04556669 (China, Hebei)	CD22 + PD-L1	Advanced malignant solid tumors	I	30 (est.)	2020	Pending Est. compl.: 2025	
NCT05518253 (China, Zhejiang)	CD70	CD70-positive advanced/metastatic solid tumors	I	36	2022	Pending Est. compl.: 2025	
NCT05468190 (China, Henan)	CD70	Advanced/metastatic CD70-positive solid tumors	I	48	2022	Pending Est. compl.: 2025	

CR = complete response, PR = partial response, SD = stable disease, PD = progressive disease, SA = severe adverse events, term = terminated, est = estimated, compl = completion.

**Table 2:** TCR-T cell-based clinical trials against cervical cancer.

Trial No. (Location)	Target	Condition	Phase	Patient No.	Start	Status	Results
NCT02280811 (United States, Maryland)	E6	HLA-A*02:01-positive HPV-16-associated metastatic/recurrent cancer	I/II	12	2014	Compl. (2016)	CR: 0 PR: 2 DLT: 0 SA: 5
NCT02153905 (United States, Maryland)	MAGE-A3	HLA-A 01-positive metastatic/recurrent cancer expressing MAGE-A3	I/II	3	2014	Compl. (2018)	CR: 0 PR: 1 DLT: 1 SA: 2
NCT02111850 (United States, Maryland)	MAGE-A3	(HLA)-DP0401/0402-positive metastatic/recurrent cancer expressing MAGE-A3-DP4	I/II	21	2014	Compl. (2021)	CR: 1 PR: 3 SD: 0 PD: 16
NCT02858310 (United States, Maryland)	E7	HLA-A*02:01-positive HPV-16-associated metastatic/recurrent cancer	I/II	180 (est.)	2017	Pending Est. compl.: 2026	
NCT03578406 (China, Chongqing)	E6 + PD-1	Metastatic/recurrent HPV-16-positive cancers	I	20	2018	Pending	
NCT05357027 (China, Chongqing)	E6	HLA-A*02- and HPV16-positive metastatic/recurrent positive cervical cancer	I/II	18	2022	Pending Est. compl.: 2024	
NCT05122221 (China, Henan)	E7	HLA-A*02- and HPV16-positive cancer	I	12	2022	Pending Est. compl.: 2024	

CR = complete response, DLT = dose-limiting toxicities, PR = partial response, SD = stable disease, PD = progressive disease, SA = severe adverse events, est = estimated, compl = completion.
